# Transcriptome analysis of *Aspergillus oryzae* RIB40 under chemical stress reveals mechanisms of adaptation to fungistatic compounds of lignocellulosic side streams

**DOI:** 10.1186/s13068-025-02688-5

**Published:** 2025-08-08

**Authors:** Miika-Erik Korpioja, Emmi Sveholm, Adiphol Dilokpimol, Tanja Paasela, Andriy Kovalchuk

**Affiliations:** VTT Technical Research Centre of Finland Ltd, Tekniikantie 21, 02150 Espoo, Finland

**Keywords:** Industrial side stream, Lignocellulosic inhibitors, Detoxification, Furaldehydes, Weak acid, Phenolics, Oxidoreductases, Transporter proteins, Filamentous fungi, Transcriptome

## Abstract

**Background:**

Industrial lignocellulosic side streams are considered an attractive carbon source for the cultivation of biotechnologically important fungi, although the presence of toxic pretreatment by-products is a major challenge yet to be overcome. *Aspergillus oryzae* is a filamentous fungus with a large secretion capacity, high tolerance for toxins, and a wide substrate range, making it a promising candidate for side stream utilization. In the present study, the cellular mechanisms of tolerance against furfural, 5-hydroxymethylfurfural (HMF), levulinic acid, ferulic acid, and vanillin were studied at the transcriptome level.

**Results:**

*A. oryzae* RIB40 was grown in the presence of different inhibitors commonly found in lignocellulosic side streams, and RNA sequencing was utilized to investigate the transcriptomic changes in response to the inhibitors. Analysis of the transcriptomic response in all conditions indicates that a large fraction of differentially expressed genes responded to the inhibitor-induced formation of reactive oxygen species (ROS). Apart from levulinic acid, all inhibitors showed strong initial suppression of metabolic pathways related to cell cycle, ribosome functions, protein folding, and sorting in the endoplasmic reticulum. Genes associated with cellular detoxification, namely, NAD(P)H-dependent oxidoreductases and efflux transporters, such as the ATP-Binding Cassette (ABC) transporters and major facilitator superfamily (MFS) transporters, showed strong upregulation upon exposure to the inhibitors.

**Conclusions:**

The results obtained provide important insights into the stress response of *A. oryzae* to the xenobiotic compounds and their cellular detoxification. Aldehydic inhibitors, especially HMF, caused a strong and severe stress response in *A. oryzae* RIB40. Additionally, we identified several highly upregulated uncharacterized genes upon exposure to the inhibitors. These genes serve as promising targets for strain engineering to build robust industrial strains capable of utilizing lignocellulosic side streams as feedstock.

**Supplementary Information:**

The online version contains supplementary material available at 10.1186/s13068-025-02688-5.

## Background

Lignocellulose is a renewable resource that can be used as an inexpensive raw material in biorefineries for the production of biofuels and other biobased chemicals, primarily by the yeast *Saccharomyces cerevisiae* [1]. Filamentous fungi, including members of the genus *Aspergillus,* have shown promise as potential alternative organisms for lignocellulose bioconversion [2–5]*. Aspergillus oryzae* is a filamentous fungus that has been used for centuries in food fermentation in Asia. Presently, *A. oryzae* serves as an important host organism in biotechnology, tolerating high concentrations of acetate and other inhibitory compounds formed during the pretreatment of biomass [6–8].

Biomass pretreatment is required to overcome the recalcitrant nature of lignocellulose, since it improves the accessibility of carbohydrate polymers for enzymatic hydrolysis [1]. However, the pretreatment process yields inhibitory by-products, including furaldehydes, phenolics, and weak acids, the composition and ratio of which depend on the type of biomass and pretreatment method applied [9]. In eukaryotes, the metabolism of xenobiotics often takes place in three phases. Phase I involves enzymatic modification of the toxic compound by oxidative enzymes such as alcohol and aldehyde dehydrogenases, reductases, cytochrome P450s, and monooxygenases [10, 11]. In phase II, they are conjugated with glutathione or sugar moiety by conjugating enzymes (e.g., glutathione S-transferases (GST) and UDP-glucuronosyltransferases (UGT)) [12] and excreted in phase III by transporters like ATP-binding cassette (ABC) or major facilitator superfamily (MFS) transporters either outside of the cell or into vacuoles [13, 14]. Alcohol and aldehyde dehydrogenases have been shown to be involved in the detoxification of hydrolysate inhibitors in *S. cerevisiae, Scheffersomyces stipitis, Neurospora crassa,* and *Pleurotus ostreatus* [2, 3, 15, 16]. In *S. cerevisiae,* the upregulation of pleiotropic drug resistance (PDR) genes has been observed under furaldehyde-induced stress. Exposure to hydrolysate inhibitors also resulted in the upregulation of ABC transporters in several fungal species [17–22], indicating the potential role of those transporters in the inhibitor tolerance. ABC transporters excrete a wide range of exogenous and endogenous toxic compounds (drugs, secondary metabolites, organic acids, etc.), and certain groups of ABC transporters are referred to as multidrug resistance (MDR) or PDR transporters [23]. Notably, *A. oryzae* possesses one of the highest numbers of predicted ABC transporters among filamentous fungi [24].

Two furaldehydes, furfural and 5-hydroxymethylfurfural (HMF), are the main furan inhibitors in lignocellulosic hydrolysates. They are formed during the pretreatment via the dehydration of pentose and hexose sugars, respectively [1]. Furaldehydes have been shown to induce reactive oxygen species (ROS) formation in *S. cerevisiae,* causing severe oxidative stress [25]*.* Furfural also inhibits enzymes involved in the central metabolism of *S. cerevisiae* and other yeasts in vitro [26, 27]. Furfural and HMF degradation pathways have been elucidated in the bacterium *Cupriavidus basilensis* [28]. Both pathways converge at 2-furoic acid, which is further converted to 2-oxoglutaric acid and then metabolized via TCA pathway. Several fungi are able to catabolize furfural and HMF, and a few of them can use furaldehydes as a sole carbon source [29]. The biochemical pathways of furaldehyde catabolism in fungi have not been well characterized, but it is hypothesized to occur similarly to that in bacteria [28, 30]. Microorganisms that are unable to catabolize furaldehydes, e.g., *S. cerevisiae*, minimize the toxicity of furaldehydes by reducing them to less toxic furfuryl alcohols [4, 16, 31–33]. In those organisms, furfuryl alcohols remain as dead-end products [34]. Reduction of furaldehydes also occurs in organisms that can catabolize them. Presumably, the reduction and oxidation processes of furaldehydes coexist in these organisms to minimize their negative impact by keeping their intracellular concentration below the toxicity limit [28, 29]. Lignin residues are the main source of aromatic compounds in lignocellulosic hydrolysates. The variety and toxicity of these aromatic compounds depend on the source material [1]. Examples of aromatic compounds occurring in lignocellulosic hydrolysates are ferulic, gallic, and syringic acids and vanillin. Generally, the aromatic compound metabolism in fungi converges to a small number of central intermediates through several metabolic pathways. Additionally, several enzymes have been identified in *Aspergillus niger* through which various aromatic compounds are metabolized into the central intermediates and ultimately into 3-oxoadipate [35–38]. Aromatic compound catabolism has also been characterized in *S. cerevisiae* [39]. Acetic acid, formic acid, and levulinic acid comprise the most abundant of the weak acids found in the lignocellulolytic hydrolysates [1]. The weak acids in the hydrolysate affect the cells through two main mechanisms. Firstly, undissociated acids can enter the cells via passive diffusion, after which they dissociate in the cytosol, decreasing the intracellular pH [40]. Simultaneously, acids induce ROS accumulation, which can lead to irreversible DNA damage and ultimately cell death [41]. Fungi combat weak acid-induced oxidative stress with several mechanisms, including scavenging ROS by glutathione, enzymatic conjugation of oxidized compounds by GSTs and UGTs, and exporting the acids through efflux transporters [12]. ABC transporters Pdr12 in *S. cerevisiae* and ABC40 in *Penicillium chrysogenum* have been shown to be involved in weak acid detoxification [19, 20, 22].

In this study, we aimed to provide insight into the transcriptional response of *A. oryzae* to common inhibitory compounds in lignocellulosic hydrolysates and identify tolerance-related genes. We produced transcriptome data under exposure to furfural, HMF, ferulic acid, vanillin, and levulinic acid and identified several highly expressed uncharacterized oxidoreductases and transporters, which could be targets for improving fungal tolerance against lignocellulose-derived inhibitors.

## Materials and methods

### Microbial strains, chemicals, media, and cultivation

The *A. oryzae* RIB40 strain was cultivated in Czapek-Dox (CZ) medium containing 3 g/L NaNO_3_, 1 g/L KH_2_PO_4_, 0.5 g/L KCl, and 0.5 g/L MgSO_4_·7H_2_O in distilled deionized water (DDIW) with the pH adjusted to 6.0 with NaOH before autoclaving. After autoclaving 1 mL/1000 mL 1000 × trace elements (1 g/L FeSO_4_·7H_2_O, 10 g/L EDTA, 4.4 g/L ZnSO_4_·7H_2_O, 2.2 g/L H_3_BO_3_, 1 g/L MnCl_2_·4H_2_O, 0.32 g/L CoCl_2_·6H_2_O, 0.32 g/L CuSO_4_·5H_2_O, 0.22 g/L (NH_4_)_6_Mo_7_O_24_·4H_2_O) were added. Solid media were supplemented with 15 g/L agar. For the preparation of spore suspensions, *A. oryzae* was grown on potato dextrose agar plates for 5 days, and the spores were collected into a solution containing 0.8% NaCl, 0.025% Tween 20, and 20% glycerol, and the resulting suspensions were stored at − 80 °C. Liquid cultures were grown at 28 °C with 230 rpm shaking in 50 mL volume in 250 mL flasks. For chemical sensitivity testing, the following chemicals were obtained from Sigma-Aldrich (Saint Louis, MO) unless otherwise mentioned: cinnamic acid, ferulic acid, furfural (FLUKA, Charlotte, NC), gallic acid, 5-hydroxymethylfurfural (Merck, Darmstadt, Germany), levulinic acid, salicylic acid, syringic acid, and vanillin.

### Chemical sensitivity

For chemical sensitivity screens, a total of 10^3^ *A. oryzae* RIB40 spores in 2 µL were inoculated at the center of CZA + 2% fructose plates with and without chemicals. All chemicals were dissolved in DMSO and then diluted into cooled, autoclaved CZ + 2% fructose agar medium in desired concentrations. Plates were incubated at 28 °C for seven days. Plates lacking chemicals were used as controls. The percentage inhibition of the radial growth of the target fungus was determined by calculating the average colony radius (ACR) for the control plates and the plates containing the chemical, and using the formula:

$${\text{Percent inhibition }} = \, \left( {{\text{ACR}}_{{({\text{control}})}} {-}{\text{ ACR}}_{{({\text{chemical}})}} } \right)/{\text{ACR}}_{{({\text{control}})}} \times { 1}00\% .$$

Control plates were grown in quadruplicate, and three biological replicates were grown for each concentration of an inhibitor. The IC_50_ value for chemicals was determined as the point at which 50% of the growth was inhibited on day 7.

### RNA extraction and sequencing

Pre-cultures of *A. oryzae* RIB40 were inoculated with 1.5 × 10^7^ spores and were cultured overnight at 28 °C with 220 rpm in shake flasks containing 250 mL CZ with D-fructose (20 g/L). Mycelium was harvested on Miracloth and washed with CZ. Equal portions of fungal hyphae were transferred to flasks containing 50 mL CZ with D-fructose (20 g/L) and the selected inhibitors at the determined IC_50_ values (Table 1). The cultures were incubated in rotary shakers for 24 h at 220 rpm. Total RNA of three biological replicates (quadruplicate for the control) was extracted at five different time points: zero hour, one hour, three hours, five hours, and 24 h after induction. Fungal hyphae were harvested, dried between tissue paper, and snap-frozen in liquid nitrogen at each time point. Frozen hyphae were ground with a mortar and pestle. Total RNA was extracted using TRIzol reagent (Invitrogen, Thermo Fisher Scientific, Carlsbad, CA) and was purified using the RNeasy plant mini kit (Qiagen, Hilden, Germany) according to the manufacturer's recommendations. The quantity and quality of RNA were determined by the Nanodrop 2000 (Thermo Scientific) and RNA 6000 Nano chips using the Agilent 2100 Bioanalyzer (Agilent Technologies, Santa Clare, CA). RNA sequencing was performed by CeGaT GmbH (Tübingen, Germany) using the Illumina NovaSeq 6000 platform (Illumina Inc., San Diego, CA). Stranded paired-end library was produced for 83 of the total 95 samples that passed the quality check with TruSeq stranded mRNA kit (Illumina Inc., San Diego, CA) for 101 bp for both pair ends with an average Q30 value of ≥ 91.38%. The sample quality of three-hour and five-hour samples of ferulic acid and levulinic acid was below the required threshold, and those samples were excluded from the dataset.

**Table 1:** Determination of IC_50_ values for the selected inhibitors

	FF	Van	FA	LA	HMF	GA	CA	SA	SyA
IC_50_ (mg mL^−1^)	0.22	0.4	1.26	2.84	1.7	10.21	0.25	0.19	20.81
IC_50_ (mM)	2.3	2.6	6.5	24.5	13.5	60	1.7	1.4	105

IC_50_ values (in mg mL^−1^ and mM) for the selected inhibitory compounds obtained from the plate assays. The IC_50_ value for the chemicals was determined as the point where 50% of the growth was inhibited on day 7. FF, furfural; Van, vanillin; FA, ferulic acid; LA, levulinic acid; HMF, hydroxymethylfurfural; GA, gallic acid; CA, cinnamic acid; SA, salicylic acid; SyA, syringic acid

### RNA-seq data analysis

RNA-seq data were analyzed using the bioinformatic toolbox at the Chipster virtual interface at the IT Center for Science (CSC), Finland [42]. Adapters were trimmed with Skewer (version 0.2.2) [43], and low-quality sequencing reads were trimmed using Trimmomatic (version 0.33) [44]. The quality of the sequencing data was checked using FastQC [(https://www.bioinformatics.babraham.ac.uk/projects/fastqc/)]. Trimmed paired-end sequencing reads were mapped to the *A. oryzae* RIB40 [8] genome retrieved with GFF from EmsemblFungi [45] using HISAT2 (version 2.2.1) [46]. BAM files were used as input files to quantify the number of short reads with HTSeq (version 0.6.1) [47], which resulted in the aligned read counts for all sequenced gene transcripts. Differential expression analysis was performed using the DESeq2 (version 1.26.0) [48]. Genes with an adjusted *p* ≤ 0.01 and log_2_ fold change of ≥ 1 or ≤  − 1 were considered as significantly differentially expressed. Individual genes were characterized by homology search using the NCBI BLASTp tool (https://blast.ncbi.nlm.nih.gov/Blast.cgi), annotations of Joint Genome Institute genomics resource database https://genome.jgi.doe.gov and the Blast2GO suite in OmicsBox [49]. The functional categories of the GO terms of the differentially expressed genes were determined using OmicsBox Blast2GO suite by setting the sequence filter at 5% of the gene set size and the GO term level at 3 [49]. The dataset supporting the conclusions of this article is available in NCBI’s Gene Expression Omnibus and is accessible through GEO Series accession number GSE296876 (https://www.ncbi.nlm.nih.gov/geo/query/acc.cgi?acc=GSE296876). Genes with adjusted *p*-value ≤ 0.01 and log_2_ fold change of ≥ 1 or ≤  − 1 in any comparison were included in the clustering analysis. Variance-stabilized transformation was performed for the normalized counts, and the average of the biological replicates was calculated for each sample and time point. Samples were divided into aldehydes (control, HMF, furfural, and vanillin) and acids (control, ferulic acid, and levulinic acid) based on the main reactive functional group. Genes were soft-clustered into 25 groups using the R package Mfuzz v2.64.0 [50] and assigned to the cluster in which they had the highest membership value. Over-representation analysis was performed in R with clusterProfiler v4.12.6 [51, 52], using enricher function for GO terms and enrichKEGG function for KEGG pathways. GO term annotations were generated for *A. oryzae* RIB40 using OmicsBox [49]. Statistical significance was assessed with *p*-value and *q*-value cut-offs of 0.01. GO.db v3.19.1 was used to retrieve GO term names, and rrvgo v1.16.0 was used to identify the corresponding parent terms for GO terms using the closest supported organism, *Saccharomyces cerevisiae* (org.Sc.sgd.db v3.19.1) [53, 54]. Results for the differential expression analysis and soft-clustering analysis are shown in Additional files 2 and 3.

### Sequence similarity network (SSN)

Sequences for the SSN were retrieved from the UniProt database, using reviewed oxidoreductase sequences with confirmed protein-level existence, within the kingdom of fungi. For each condition, 30 of the most highly upregulated oxidoreductases were selected. After removing duplicates, 91 *A. oryzae* amino acid sequences of interest were combined with the oxidoreductase sequences retrieved from UniProt, resulting in 1519 amino acid sequences (Additional file 4). SSN was generated using SSNpipe, which is freely available on GitHub (https://github.com/ahvdk/SSNpipe), and the edge threshold cut-off was set to 10^–60^. The SSNs were visualized and edited in Cytoscape v3.10.1 using the yFiles organic layout [55].

## Results and discussion

### IC_50_ determination of nine growth inhibitors for *A. oryzae* RIB40

**Fig. 1:**
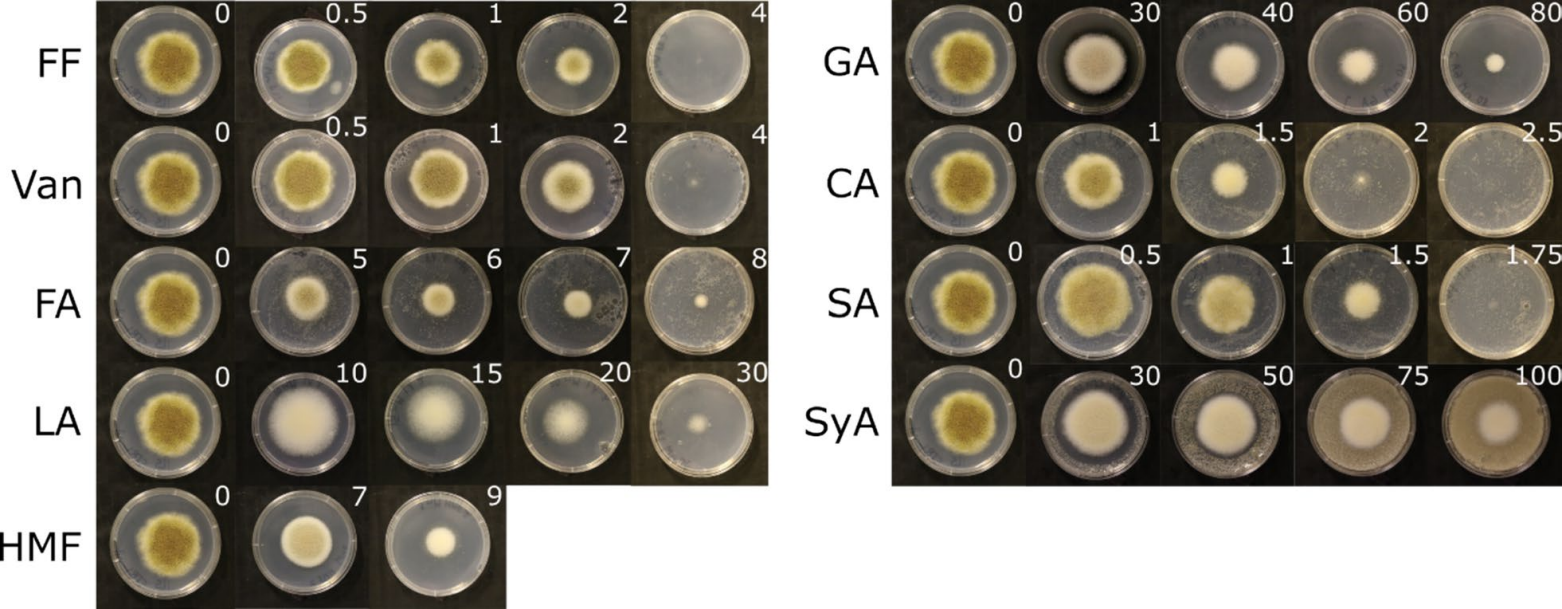
Determination of IC_50_ values for the selected inhibitors. Growth profiles of *Aspergillus oryzae* RIB40 on solid media supplemented with different inhibitors found in lignocellulosic hydrolysates. Cultures were grown on CZA medium supplemented with 2% D-fructose and increasing concentrations of the inhibitory compounds. Plates were incubated at + 28 °C for seven days. Control plates without supplementation of the inhibitors were made in quadruplicate. Three biological replicates were prepared for each concentration of the inhibitor. The white number in the upper right corner of each plate indicates the inhibitor concentration (mM). FF, furfural; Van, vanillin; FA, ferulic acid; LA, levulinic acid; HMF, hydroxymethylfurfural; GA, gallic acid; CA, cinnamic acid; SA, salicylic acid; SyA, syringic acid

Lignocellulosic hydrolysates contain three main classes of compounds that have an inhibitory effect on fungal growth, i.e., furaldehydes, phenolics, and organic acids [31]. Nine compounds representing those three groups were selected based on literature data. Growth profiles on solid media with and without the inhibitors were generated and analyzed to evaluate the effect of the selected compounds on the growth of *A. oryzae* strain RIB40 (Fig. 1**, **Table 1). Of the compounds tested, salicylic acid, cinnamic acid, vanillin, and furfural showed the strongest inhibitory effect. In contrast, syringic acid and gallic acid were the least effective and were excluded from further study. In addition, because of their low solubility in aqueous solutions, cinnamic acid and salicylic acid were also excluded from further study. Therefore, five compounds, i.e., ferulic acid, furfural, HMF, levulinic acid, and vanillin, were selected for transcriptome analysis in shake flask cultivations and were subsequently cultivated at their determined IC₅₀ values (Table 1).

### Transcriptional response of *A. oryzae* to growth-inhibiting compounds

Sequencing of RNA samples that passed quality control generated approximately 44 million 101 bp paired-end reads per sample. Sufficient quality RNA-seq data from levulinic and ferulic acid samples at three- and five-hour time points could not be generated; therefore, those data points were excluded from further analysis. The Q30 values for all remaining samples were higher than 91.4%, and overall alignment rates per sample against the *A. oryzae* RIB40 genome [8] were between 83.2% and 94.8%, the average being 90.5%. Genes with adjusted *p*-value < 0.01 and Log_2_ fold change (LFC) > 1 or < -1 were considered to be significantly differentially expressed genes (DEG) (Table 2; Tables S1–S5). Principal component analysis (PCA) was conducted to evaluate the reliability of the sequencing data and the replicate samples (Fig. S1). The first two components of the PCA of the dataset, which include all five time points and six conditions, accounted for 78% of the variation, indicating that time and the presence of inhibitors were the main factors contributing to the observed variation between the samples. Replicate samples generally clustered closely together in the PCA plot, except for the HMF 24-h samples, where one outlier sample was observed, but was still included in the dataset.

**Table 2:** Number of differentially expressed genes for each inhibitor and time point

	1 h		3 h		5 h		24 h	
	Up	Down	Up	Down	Up	Down	Up	Down
Furfural	1660	2277	751	604	254	85	75	5
HMF	2356	2718	2004	1868	1773	955	319	99
Vanillin	1816	1941	920	461	198	126	80	10
Ferulic acid	2340	2333	N. D.	N. D.	N. D.	N. D.	2249	2889
Levulinic acid	1518	1981	N. D.	N. D.	N. D.	N. D.	2104	2446

N.D., not determined

**Fig. 2:**
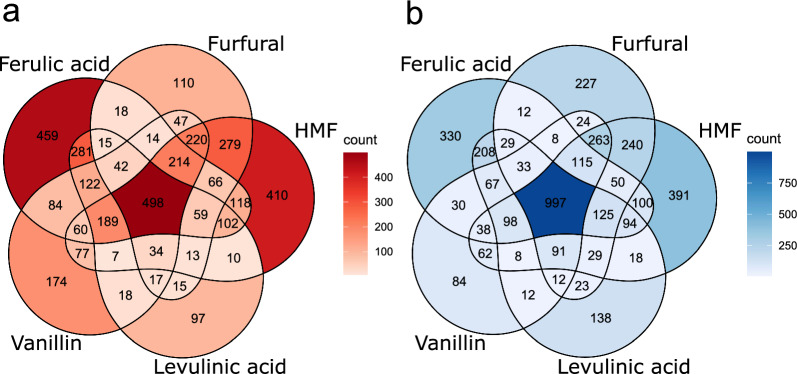
Venn diagrams of differentially expressed genes of each inhibitor one hour after induction (**a**) upregulated genes (**b**) downregulated genes

All five conditions shared 691 genes throughout all the time points that were upregulated and 1131 genes that were downregulated. However, there was a clear difference between the tested compounds in the temporal pattern of the transcriptional response. The highest numbers of DEGs, both up- and downregulated for furfural, HMF, and vanillin, were observed one-hour post-exposure (Table 2), gradually decreasing over time, with the least changes at 24 h after induction. Compared to furfural and vanillin, HMF induced a stronger and longer-lasting transcriptional response, reflected in a greater number of DEGs at later time points. In contrast, the total number of DEGs on ferulic acid and levulinic acid increased between one hour and 24 h. Venn diagrams of DEG detected one hour after the addition of inhibitors show that 498 out of 3869 genes (12.9%) were induced on all five compounds (Fig. 2a), whereas 997 out of 3956 genes (25.2%) were downregulated on all five compounds (Fig. 2b). There was also a high overlap in responses to chemically or functionally related compounds, i.e., between two furaldehydes (furfural and HMF) and between two aromatic compounds (ferulic acid and vanillin). Thus, there were 279 upregulated and 240 downregulated genes that responded exclusively to furaldehydes (furfural and HMF), whereas 281 upregulated and 208 downregulated genes responded specifically to two weak acids (ferulic acid and levulinic acid). A substantial fraction of genes also responded to compounds with aldo groups (furfural, HMF, or vanillin) but not to organic acids (220 upregulated and 263 downregulated genes, respectively). Finally, a set of 115 upregulated genes was shared between all tested compounds except for levulinic acid. Significant overlap in the downregulated genes suggests that a common cellular response to stress is activated in response to any inhibitor treatment applied. On the other hand, the lower overlap among upregulated genes implies that each inhibitor induced a rather specific transcriptional response, which is likely tailored to address the unique challenges posed by each compound.

GO analysis was used to identify the most abundant functional groups among DEGs. Our analysis revealed a considerable overlap in GO terms between up- and downregulated genes (**Fig. S2**). Such GO terms as “Small molecule binding,” “Oxidoreductase activity,” “Hydrolase activity,” and “Transferase activity” had high scores among upregulated as well as among downregulated genes on all inhibitors and at all time points. This result might indicate large-scale metabolic rearrangements in cells exposed to chemical stress. Additionally, genes assigned to the GO term “Transmembrane transport” had high scores in most of the conditions, reflecting the importance of transporter proteins in cell adaptation to chemical stress.

Our data suggest that exposure to the fungistatic compounds triggers an initial shock response in the cells. This shock response occurs very fast (within the first hour) and affects about 30% to 40% of the total number of genes. At the later time points, on the aldehydic inhibitors, the total number of DEGs decreased, and the number of upregulated genes (especially genes associated with the detoxification of the inhibitors) exceeded the number of downregulated genes, indicating that the cells are beginning to recover from the inhibitor-induced stress.

### Clustering-based analysis of the transcriptional responses to inhibitory compounds

To explore broader transcriptional trends beyond GO term analysis, soft clustering was performed on the differentially expressed genes, enabling the identification of distinct expression patterns within the dataset. Enriched GO terms and KEGG pathways for each cluster are listed in Tables S6 and S7, and cluster-specific information is in Additional file 3.

Transcriptional regulation appears to differ in response to aldehydes versus acids. Upon exposure to aldehydic compounds, especially furaldehydes, clusters 10 and 17 (Fig. S3), which contain genes related to transcriptional regulator activity, show an initial strong upregulation, with expression levels gradually returning to baseline over time. In contrast, for the acids, in clusters 22 and 23 that contain genes related to transcriptional activity, the expression of the related genes was significantly lower in comparison to the control (Fig. S4). Differences between the aldehyde and acid compounds regarding transcriptional regulator activity might indicate distinct cellular detoxification strategies, possibly due to the acidic compounds' lower reactivity and reduced capacity to cause cellular damage, in contrast to the aldehydes. For aldehyde clustering, genes in clusters 8 and 23 displayed a strong initial upregulation, especially on HMF. Cluster 23 consists of genes associated with both proteasomal ubiquitin-independent and -dependent protein catabolic processes, and cluster 8 genes are associated with terms related to autophagy and peroxisomes. Strong upregulation of proteasome-related genes in cluster 23 suggests a cellular response to combat ROS-induced protein damage, whereas the upregulated genes related to autophagy in cluster 8 further support this coordinated response to oxidative stress, since autophagy is important for oxidative stress tolerance in fungi and is likely induced by the increase of intracellular ROS levels [56–58]. Peroxisomes generate ROS naturally through several metabolic functions, and in the plant pathogen *Alternaria alternata* autophagy-mediated degradation of peroxisomes was found to be a central response mechanism to high levels of ROS [58].

In both cluster analyses, genes associated with cell cycle progression, chromosome regulation, and DNA replication (clusters 2 and 3 for aldehydes, 24 and 25 for acids) are strongly downregulated in the first hours following exposure to inhibitory compounds. This suggests that the cell cycle is rapidly halted, likely as a protective response to oxidative stress, which is known to induce cell cycle arrest in *S. cerevisiae* [59]. Suppression of these processes likely serves a dual function: preventing genomic instability caused by ROS generated during inhibitor metabolism while conserving energy, as metabolism shifts toward catabolic and stress adaptation pathways. At the same time, on aldehydes clusters 4, 15, and 16, which are enriched with GO and KEGG terms related to transcription, translation, RNA metabolism, protein packing, and sorting in the ER and Golgi, are strongly downregulated one hour after induction. Expression levels on clusters 15 and 16 recover to the control strains' level by the fifth hour, except for HMF, but cluster 4 genes show strong upregulation at three and five hours after induction for furfural and HMF, respectively. The samples exposed to ferulic acid behave similarly (clusters 12, 14, and 15), but interestingly, for levulinic acid, only ER-related terms were downregulated one hour after induction, in contrast to RNA metabolism and ribosome-related terms that were upregulated moderately. Similar results for ribosomal gene downregulation have been reported for *S. cerevisiae* under lignocellulose-derived inhibitor stress [21, 60, 61].

Cluster 6 for aldehydes and 1 and 8 for acids, associated with oxidoreductase activity terms, showed upregulation in comparison to the control conditions, although on aldehydes, the upregulation was very modest, except for HMF. However, on closer inspection, the majority of the most highly expressed oxidoreductases did not cluster with the aforementioned clusters, but instead into clusters 18 and 9 for aldehydes and acids, respectively, although neither cluster is associated with oxidoreductase activity terms, although both clusters display a strong relative expression change upon first hours after induction.

The initial response is most potent in cells exposed to the furaldehydes and ferulic acid. However, furfural-treated cells recover much faster than those exposed to HMF, suggesting that HMF imposes a more prolonged or severe metabolic burden on the fungus. The clustering analyses suggest that aldehydic compounds generate a much stronger stress response in *A. oryzae*, during which cellular functions are halted, and energy is conserved as the cells focus on detoxification and recovery of homeostasis. Levulinic acid appears to provoke a distinct cellular reaction compared to furaldehydes or aromatic compounds, as genes involved in RNA metabolism and ribosomal functions are upregulated upon exposure to levulinic acid, contrary to the effect of all the other inhibitors. Additionally, oxidoreductase activity is upregulated only moderately on levulinic acid compared to other inhibitors. Following these results, we focused our further analysis on oxidoreductase genes and genes encoding ABC and MFS transporters.

### Phase I detoxification is mainly driven by NAD(P)H-dependent oxidoreductases in *A. oryzae*

Two principal mechanisms of cell tolerance against inhibitory compounds are their metabolic transformation and excretion/sequestration [12]. Our data showed differential expression of hundreds of predicted oxidoreductase (EC 1.-.-.-.) genes that potentially contribute to phase I detoxification, i.e., the metabolic transformation of the lignocellulose-derived inhibitors. Both clusters 18 for aldehydes and 9 for acids contained largely overlapping oxidoreductases, which were highly induced after the first hour of induction. The majority of the oxidoreductases were putative alcohol and aldehyde dehydrogenases (ADH, ALDH), cinnamyl alcohol dehydrogenases (CAD), aldo–keto reductases (AKR), trans-enoyl reductases (TER), and aryl alcohol dehydrogenases (AADH). These enzymes catalyze the NAD(P)H-dependent interconversion of alcohols, aldehydes, and ketones, as well as the reduction of C=C double bonds in enoyl-CoA substrates. Additionally, several short-chain dehydrogenase/reductases (SDR) genes were observed to be highly induced. Many of these enzymes have been shown to be involved in the detoxification of various inhibitors found in the lignocellulose hydrolysate in several organisms [2, 16, 33, 62]. Additionally, among the most highly expressed oxidoreductases were also glucose-methanol-choline oxidoreductases (GMC), peroxidases, cytochrome P450 monooxygenases (CYP), and fatty acid desaturases (Table S8).

**Fig. 3:**
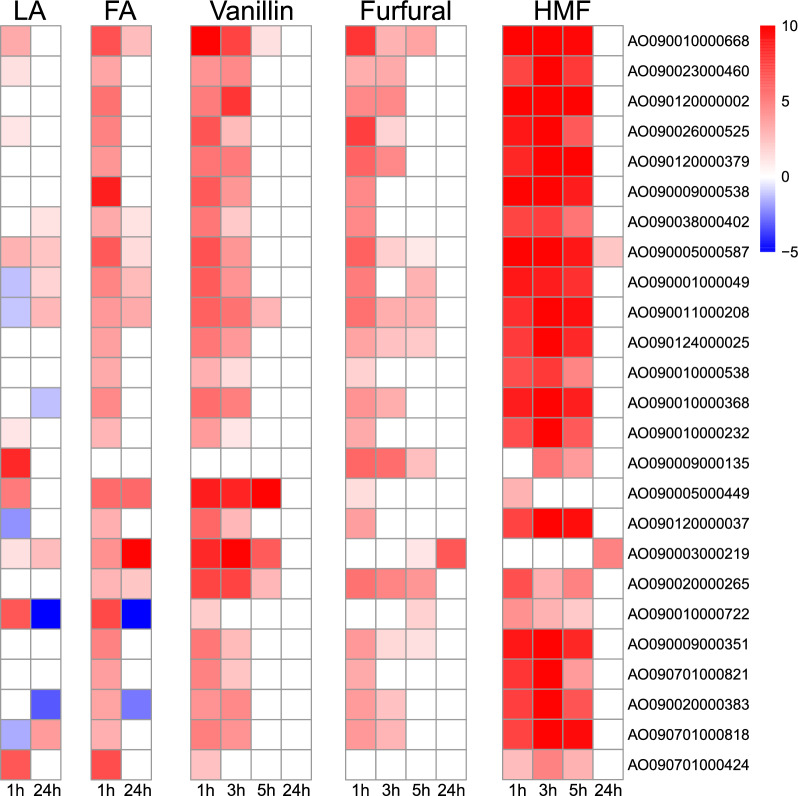
Heatmaps of LFC values of the most upregulated oxidoreductases one hour after induction under levulinic acid (LA), ferulic acid (FA), vanillin, furfural, and HMF in comparison to the control strain at different time points. LFC values in the heatmaps are capped from − 5 to 10

The strongest initial induction of the putative oxidoreductase genes was observed on HMF, where several oxidoreductases were upregulated during the first five hours after induction with an LFC over 10 (Fig. 3, Table S8). Putative CAD AO090010000668 was the gene with the highest fold change one hour after induction on vanillin, with an LFC of 10.7, and the gene with the second highest fold change on furfural, with an LFC of 8.0. Upregulation remained high on HMF even five hours after induction, when increased expression of a 9.8 LFC was still observed. Similarly, on levulinic and ferulic acid, AO090010000668 was among the most highly upregulated oxidoreductases one hour after induction. Three putative AADHs, AO090120000002, AO090026000525, and AO090120000379, exhibited strong induction in response to all inhibitors except for levulinic acid. On HMF, AO090120000002 showed the most substantial induction, as the upregulation reached LFCs of 16.1 and 13.1 three and five hours after induction, respectively. The putative AKR AO090009000538 was the most upregulated gene on HMF and the most upregulated oxidoreductase on ferulic acid one hour after induction, and among the most expressed oxidoreductases on furfural and vanillin. ALDH AO090009000135 was the most upregulated oxidoreductase on levulinic acid after one hour, with an LFC of 8.4. It also showed high upregulation on furaldehydes, but not on aromatics.

**Fig. 4:**
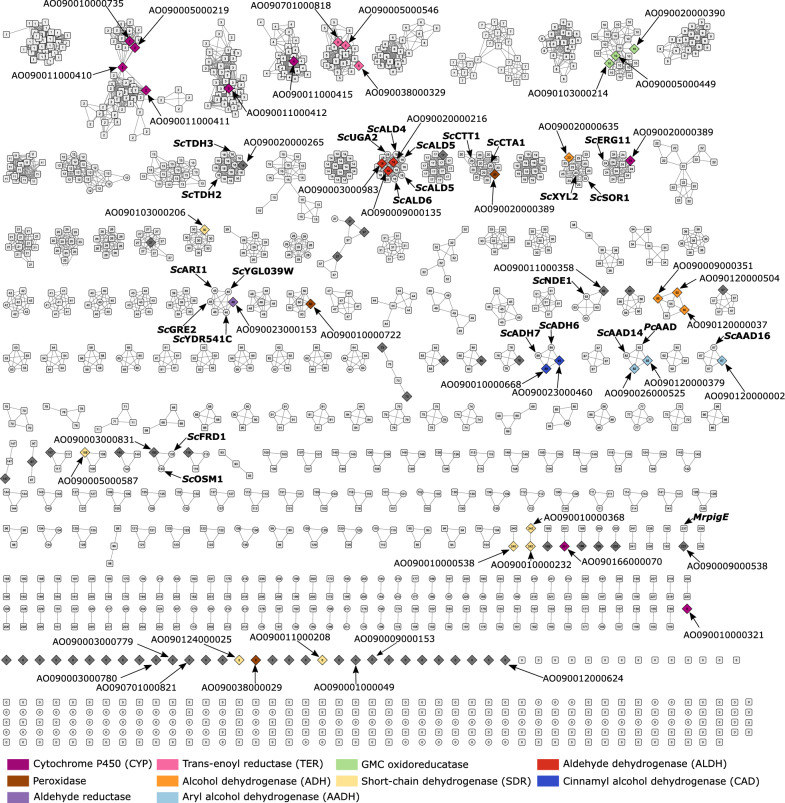
Sequence similarity network with an E-value cut-off of 10^–60^ for reported fungal amino acid sequences of oxidoreductases (EC: 1.-.-.-). The sequences were colored based on characterized activity; the shape distinguishes the *A. oryzae* sequences (diamond) from sequences of characterized *S. cerevisiae* (circle) and other fungal proteins (square). Bolded sequences represent genes primarily involved in chemical stress responses, with some included to provide functional context within the clusters. *Sc*, *Saccharomyces cerevisiae*; *Pc, Phanerochaete chrysosporium*; *Mr, Monascus ruber*

The amino acid sequences of the most strongly upregulated oxidoreductases for each inhibitor were used to build a sequence similarity network (SSN). SSN can be used to cluster and visualize similarities within large enzyme families to determine the structural and functional relationships of the enzyme sequences [63] (Fig. 4). Both *A. oryzae* CADs clustered in the SSN together with *S. cerevisiae* CADs *ADH6* and *ADH7* suggesting that they may be functionally related to those putative yeast CADs. Both *ADH6* and *ADH7* have been shown to have substrate specificity toward aromatic compounds and aliphatic aldehydes, which are both found in the lignocellulolytic hydrolysates and have been shown to contribute to the tolerance toward furaldehydes by reducing furfural and HMF to less toxic furfuryl alcohol and HMF alcohol, respectively [15, 33, 62, 64]. Additionally, ADH6 has been shown to reduce vanillin in *S. cerevisiae* [65]*. A. oryzae* AADHs formed two distinct clusters in the SSN, where AO090120000002 clustered together with *S. cerevisiae AAD16,* whereas AO090120000379 and AO090026000525 clustered together with *Phanerochaete chrysosporium* AADH *AAD1* and *S. cerevisiae AAD14*. It has been shown that recombinant *P. chrysosporium* AADH Aadp1 has substrate specificity toward HMF and vanillin [66]. AADHs act primarily on aromatic alcohols and aldehydes [67], but they have been shown to be upregulated under furaldehyde-induced stress in *P. ostreatus* and *S. cerevisiae,* including *AAD14* and *AAD16* [2, 60]. Putative aldehyde reductase AO090023000153 was strongly upregulated on HMF (Table S9) and in the SSN, it clustered together with *S. cerevisiae* aldehyde reductases *ARI1, GRE2, YGL039W,* and *YDR541C,* all of which have been shown to reduce both furfural and HMF, among other aldehydes found in hydrolysates, suggesting a similar role in *A. oryzae* [68–71]. However, the function and involvement of many highly expressed oxidoreductases, which are presumably involved in detoxification, remain unclear, as they do not cluster together with any previously characterized fungal oxidoreductase in the SSN.

In response to each inhibitor, peroxidases were upregulated early after induction, likely to combat the inhibitor-induced ROS, while fatty acid desaturases were upregulated at later time points to restore cell membrane composition after lipid peroxidation damage to maintain membrane fluidity. Surprisingly, few CYPs exhibited significant upregulation during the initial hours after induction, despite their well-documented role in xenobiotic detoxification in fungi and the fact that the *A. oryzae* genome is predicted to encode at least 149 CYPs [8, 11]. Overall, there was considerable overlap among the inhibitors regarding the most highly expressed oxidoreductases, like the CAD AO090010000668 and AADHs AO090120000002, AO090026000525, and AO090120000379, suggesting that they may be part of a unified response to chemical stress in *A. oryzae*. Our findings indicate that the detoxification of lignocellulose-derived inhibitors in *A. oryzae* primarily relies on NAD(P)H-dependent oxidoreductase activity and aligns with previous studies on fungal AADHs and CADs, supporting their activity on cyclic compounds such as furaldehydes in addition to aryl-alcohols and highlighting their potential role in xenobiotic detoxification in fungi. Our SSN analysis can not only guide the functional verification of *A. oryzae* enzymes that are clustered together with well-known genes but also emphasizes the possibility to discover novel enzyme specificities among genes that remain outside the known clusters. However, a large proportion of the highly induced oxidoreductases, especially SDRs, remain functionally uncharacterized.

### Lignocellulose-derived inhibitors elicit distinct transcriptional responses for ABC and MFS transporters

In the phase III of xenobiotic detoxification, the harmful compounds are either excreted outside of the cells or sequestrated into vacuoles [12, 72]. In our dataset, multiple members of two transporter families, MFS and ABC transporters, were upregulated in response to the treatments. The largest subset of MFS transporters clustered into cluster 12 for aldehydic compounds and into cluster 18 for acids. However, the most highly upregulated MFS transporters were distributed more evenly across the clusters, as clusters 21, 23, and 25 for aldehydes and clusters 6 and 8 for acids contained most of the highly expressed MFS transporters. ABC transporters clustered largely into different clusters than MFS transporters, but interestingly, five ABC transporters clustered in the same cluster 18 on aldehydes as the most upregulated oxidoreductases, and eight ABC transporters clustered in cluster 9 on acids.

**Fig. 5:**
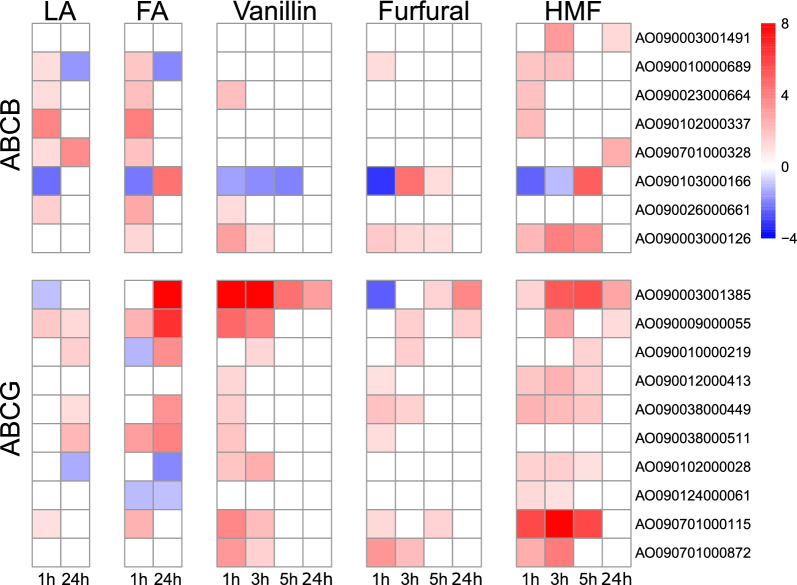
Heatmaps of LFC of the most highly upregulated *A. oryzae* ABC transporter genes under levulinic acid (LA), ferulic acid (FA), vanillin, furfural, and HMF in comparison to the control strain at different time points. LFC values in the heatmaps are capped from − 4 to 8

Eukaryotic ABC proteins are classified into nine subfamilies, of which the largest and most relevant are the ABCB, ABCC, and ABCG subfamilies [24]. Eukaryotic ABC transporters function mainly as exporters with a broad range of substrates [23]. Overall, most of the highly upregulated ABC transporters were ABCG transporters. Two ABCG transporters, AO090003001385 and AO090701000115, showed the strongest upregulation on HMF and vanillin. AO090003001385 was upregulated on vanillin with an LFC of 8.5 three hours after induction and still 24 h after induction with an LFC of 3.1, whereas on HMF, expression reached an LFC of 5.6 five hours after induction (Fig. 5, Table S9). Expression of AO090701000115 was strongest on HMF, with an LFC of 8.5 observed three hours after induction. AO090701000115 is a homolog of *atrF,* which has been associated with antifungal azole resistance in invasive aspergillosis-causing *A. fumigatus* [73]*.* Additionally, ABCG transporters AO090010000219, AO090011000378, and AO090012000328 have also been shown to contribute to azole resistance [74]. ABCG AO090009000055 displayed strong upregulation on aromatics, as an LFC of 4 was reached three hours after induction on vanillin, and an LFC of 6.5 on ferulic acid 24 h after induction. Upregulation of AO090038000511 was only observed on ferulic acid, whereas elevated expression levels for AO090038000449 were only attained under exposure to furaldehydes. In *P. chrysogenum,* Weber et al. [22] showed that a deletion of an ABCG transporter ABC40 (81% amino acid sequence similarity to AO090038000511 and 66% similarity to AO090009000055) led to increased sensitivity to phenylacetic, sorbic, and benzoic acids, inferring ABC40 involvement in weak acid detoxification. Other studies have also shown involvement of ABC transporters in resistance to several weak acids in *S. cerevisiae* [19, 20]. Interestingly, the ABCG transporters AO090003000126, AO090012000413, AO090038000449, and AO090701000115 all clustered in cluster 18 on aldehydic compounds.

Several MFS transporters were also highly upregulated under exposure to inhibitors found in lignocellulolytic hydrolysates (Table S9). The research of fungal MFS transporters has mainly focused on sugar transporters, which constitute the largest portion of the transporter family, apart from the invasive fungal species, such as those in the genus *Candida.* Many MFS transporter families are associated with drug efflux and the secretion of toxins, and several MFS transporters have been shown to confer resistance to weak acids, azoles, and other drugs in *S. cerevisiae* [75]. In *S. cerevisiae,* polyamine transporters have been shown to be upregulated under exposure to furaldehydes [21]. Of the most highly upregulated MFS transporters (**Fig. S5**), AO090010000407, AO090005001365, AO090102000388, and AO090009000008, which have been annotated as multidrug transporters in *A. flavus*, showed strong upregulation on at least four different conditions, whereas several others were upregulated on two or three conditions. Interestingly, AO090009000008, which is not putatively annotated as a polyamine transporter, showed similarity to yeast polyamine transporters Tpo2p and Tpo3p, which have been shown to alleviate acetic acid stress in *S. cerevisiae* [75]. Tpo2p and Tpo3p also showed lower similarity to several other highly induced transporters, AO090102000388, AO090102000135, AO090026000494, and AO090010000350. AO090010000407 was the most upregulated MFS transporter for the aldehydic compounds one hour after induction, and for HMF, it remained the most upregulated MFS transporter still five hours after induction, with an LFC of 9.3. For levulinic acid, the putative monocarboxylate transporters AO090020000614 and AO090038000078 displayed a significant increase in expression. AO090020000614 was the most highly induced gene on levulinic acid with an LFC of 11.6 one hour after induction, whereas AO090038000078 was upregulated with an LFC of 5.6. On the other inhibitors, considerably lower upregulation was observed for the two transporters. Additionally, putative multidrug transporter AO090038000031 showed significantly stronger upregulation only on levulinic acid, one hour after induction.

The *A. oryzae* genome is predicted to code for at least 365 MFS transporters and 66 ABC transporters alone; however, the natural substrates of these transporters involved in xenobiotic excretion remain elusive [8]. Most efflux transporters can excrete a wide range of substrates, thus complicating the identification of the role of a single transporter in detoxification. The strong induction of specific ABC and MFS transporters observed in our study likely reflects their important role in physiological responses to chemical stress. At least some of the induced transporters probably excrete either lignocellulosic inhibitors or products of their conversion, thereby helping reduce their intracellular concentrations. Our data suggest that the monocarboxylate transporter AO090020000614 is crucial for levulinic acid tolerance in *A. oryzae*.

### Identification of genes involved in aromatic compound metabolism

Four putative decarboxylases, AO090001000093, AO090001000094, AO090003000423, and AO090003000424, showed upregulation on ferulic acid, and only AO090001000093 was upregulated on vanillin (Table S10). AO090001000093 and AO090001000094 were highly upregulated one hour after induction with an LFC over 7.4. Both AO090001000093 and AO090003000424 showed amino acid similarity to *S. cerevisiae* ferulic acid decarboxylase *FDC1* and its homolog in *A. niger* [76, 77], whereas AO090001000094 and AO090003000423 are homologous to *S. cerevisiae PAD1* and *A. niger padA1* [78]. Both genes are essential for the decarboxylation of ferulic acid into 4-vinylguaiacol in *S. cerevisiae* [76]*.* It has been shown that in *A. niger* PadA1 does not decarboxylate ferulic acid but ferulic acid and other hydrocinnamic acids are degraded through CoA-dependent β-oxidative pathway [37, 76, 78]. However, the homologs of the CoA-dependent β-oxidative pathway genes in *A. oryzae* did not show differential expression in our data on ferulic acid, suggesting that *A. oryzae* may utilize different metabolic pathways in ferulic acid conversion. Our expression data, together with previous studies, suggest that AO090001000093 and AO090003000424 are responsible for decarboxylating ferulic acid into 4-vinylguaiacol in *A. oryzae,* although it is not known which genes are responsible for the conversion of 4-vinylguaiacol further in the pathway*.* A high induction level of AO090001000093 (it is the most highly induced gene on ferulic acid one hour after induction) indicates the importance of this reaction in alleviating the toxicity of ferulic acid.

In *A. niger,* vanillin is converted into 3-oxoadipate via metabolic pathway consisting of five enzymes; vanillin dehydrogenase (*vdhA*), vanillate hydroxylase (*vhyA*), methoxyhydroquinone 1,2 dioxygenase (*mhdA*), maleylacetate reductase, and 4-oxomonomethyl adipate esterase (*omeA*) [36]. *A. niger vdhA* has two homologs in *A. oryzae,* AO090003000983 and AO090005000290, but only AO090003000983 was found to be differentially expressed. Upregulation was not observed on ferulic acid. Vanillin is typically found in cells at low concentrations and is quickly metabolized [79, 80]. It is possible that the intracellular concentrations of intermediate vanillin during ferulic acid conversion may not be high enough to require strong *vdhA* activation, as observed with *A. niger* cells fed vanillin extracellularly [80]. In contrast, *vhyA* homolog AO090003000219 was heavily upregulated on both aromatics (Table S10). *MhdA* homolog AO090012000156 was upregulated on both aromatics, but with a higher LFC and longer upregulation period on vanillin. Maleylacetate reductase AO090012000939 showed upregulation on vanillin until five hours after induction, but no differential expression was observed on ferulic acid. *OmeA* homolog AO090023000186 was upregulated on both aromatic compounds, with an LFC of 5.9 on vanillin after three hours of induction. Strong upregulation of decarboxylases and *vhyA,* and the lack of differential expression of the genes present in the CoA-dependent β-oxidative pathway, suggests that ferulic acid is metabolized via the same metabolic route as vanillin, even though *vdhA* is not differentially expressed.

**Fig. 6:**
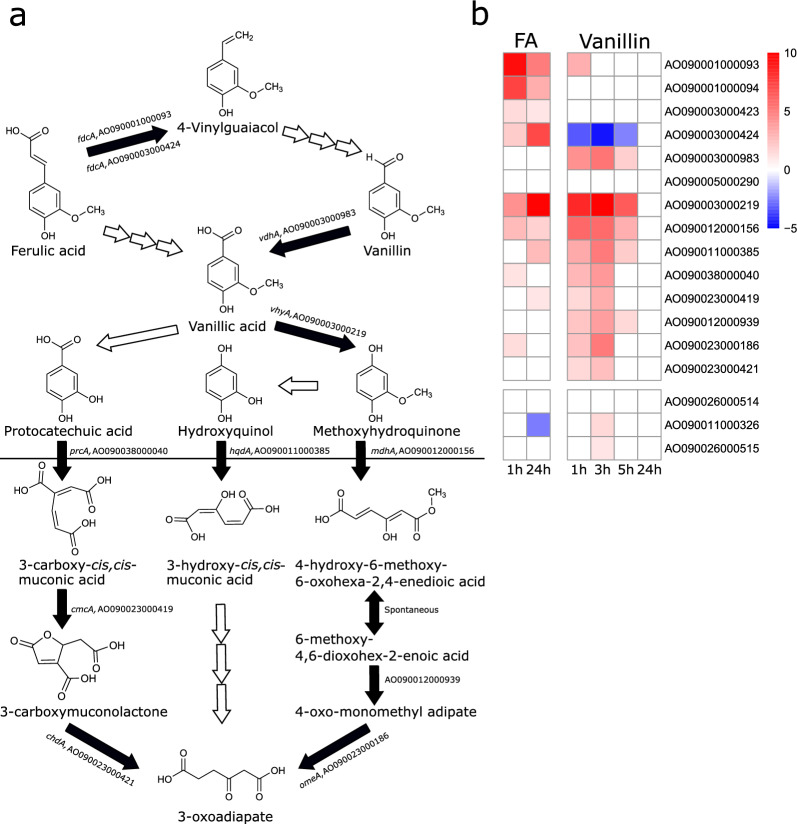
Overview of the genes proposed for ferulic acid and vanillin (**a**) metabolic pathways in *A. oryzae* (**b**) LFC values of the genes. Black arrows indicate a known homolog from *A. niger* where the corresponding genes have been characterized. White arrows indicate a suggested conversion where the corresponding genes are unknown. Multiple arrows indicate multiple-step conversions. Black line indicates ring cleavage. LFC values in the heatmaps are capped from -5 to 10. Genes after the gap belong to the CoA-dependent β-oxidative pathway

The intermediate methoxyhydroquinone could be converted into 4-hydroxy-6-methoxy-6-oxohexa-2,4-enedioic acid either by MhdA or into hydroxyquinol by an uncharacterized *O*-demethylase. However, unlike in bacteria, no fungal *O*-demethylases have been characterized in detail. Fungi also exploit cytochrome P450 oxidases, peroxidases, laccases, and ROS generated by Fenton chemistry to achieve lignin *O*-demethylation [81]. In response to vanillin treatment, *hqdA* homolog AO090011000385 displayed strong upregulation up to five hours after induction, but on ferulic acid, increased expression was only observed 24 h after induction. Even though *A. niger* HqdA has high catalytic efficiency toward hydroxyquinol [82]. Lubbers et al. [35] showed that the deletion of *hqdA* did not affect the growth on ferulic acid, vanillin, or vanillic acid, suggesting that the alternative pathway where methoxyhydroquinone would be converted into hydroxyquinol is not the primary metabolic route. Another possible pathway through which vanillin could be metabolized is the protocatechuate branch of the β-ketoadipate pathway, which would require the *O*-demethylation of vanillic acid into protocatechuic acid. However, such an enzyme has not yet been discovered in fungi. Sgro et al. [83] characterized genes involved in the protocatechuate branch of the β-ketoadipate pathway in *A. niger*, and in our data all corresponding *A. oryzae* homologs, namely, protocatechuic acid 3,4-dioxygenase *prcA* AO090038000040*,* the 3-carboxy-*cis,cis*-muconate cyclase *cmcA* AO090023000419, and 3-carboxymuconolactone hydrolase/decarboxylase *chdA* AO090023000421, displayed strong upregulation on vanillin, whereas on ferulic acid, the upregulation of genes involved in the pathway was more modest, and upregulation was not observed for all the genes. Based on our transcriptome data and previous studies by others in *A. niger,* we suggest a metabolic pathway of ferulic acid and vanillin metabolism (Fig. 6) [35, 36, 83].

### Identification of the genes involved in furaldehyde degradation

The catabolism of furaldehyde in fungi is not as well understood as that in bacteria, although a degradation pathway for HMF has been proposed in *Aspergillus nidulans* as well as for both furfural and HMF in the biodetoxification fungus *Amorphotheca resinae* ZN1 [4, 28, 29, 84]. For *S. cerevisiae*, the reduction of both furaldehydes to their respective alcohols has been demonstrated, but their oxidation into acids has not [85]. *A. resinae* ZN1 has been shown to convert both furfural and HMF into their respective alcohols and acids [84]. Ran et al. [84] also proposed a pathway for furaldehyde catabolism in *A. resinae*, suggesting that the pathway occurs similarly to that in *C. basilensis* as presented by Koopman et al. [28]. In the proposed pathway, both furaldehydes are reduced to more stable and less toxic alcohols when sufficient oxygen is not available. If oxygen is present, the inhibitors or their respective alcohols are oxidized back to aldehydes and subsequently into their respective acids. HMF is oxidized into HMF acid, which is converted to 2,5-furan-dicarboxylic acid (FDCA) and subsequently to 2-furoic acid (FCA), where the degradation pathways for HMF and furfural converge, followed by a further six-step conversion to 2-oxoglutaric acid, which then enters the TCA cycle. In a transcriptome analysis conducted by Wang et al. [29], furaldehyde-induced stress led to a significant upregulation of ADHs, ALDHs, SDRs, and AKRs in *A. resinae* ZN1. Several of the genes proposed to be involved in the detoxification of the furaldehydes, either to their respective alcohols or acids, showed strong similarity to the genes induced by the furaldehydes in *A. oryzae* (Table S11)**.**

Martins et al. [4] identified an HMF degradation pathway in *A. nidulans*, corresponding to the previously identified three-gene cluster *hmfFGH* of *C. brasiliensis,* which is responsible for the oxidation and decarboxylation steps of HMF conversion into FCA [28]. However, none of the homologs or orthogroup members of AN4212/*hmfG* displayed upregulation on HMF in our data. Both AN7164/*hmfF* and AN12147/*hmfG* are decarboxylases that should mediate the conversion of FDCA to FCA. Both genes have two homologs in *A. oryzae*: AO090001000093 and AO090003000424 for AN7164, and AO090001000094 and AO090003000423 for AN12147. Martins et al. [4] did not observe any significantly changed expression levels for AN12147 when *A. nidulans* was exposed to HMF or its derivatives. On the contrary, our data showed high upregulation for AO090003000424 and AO090003000423 in response to both furaldehydes, and for AO090001000094 in response to HMF (Table S11). As shown by both Ran et al. [84] and Martins et al. [4], the conversion rates of HMF and furfural into their corresponding acids in *A. resinae* and *A. nidulans*, even under favorable conditions, are significantly lower compared to their conversion into corresponding alcohols, which may explain the lack of strong induction for the AN4212 homologs in *A. oryzae*, if they are involved in the HMF degradation. Similarly to *A. resinae,* we have identified several genes possibly involved in the furaldehyde degradation in *A. oryzae.* However, to our knowledge, no enzyme has been characterized in fungi that can convert furfural or HMF to their respective acids or further in the proposed degradation pathway.

## Conclusions

This study describes the transcriptional response of *A. oryzae* RIB40 to cytotoxic compounds of lignocellulosic hydrolysates. Our results highlight pathways through which *A. oryzae* metabolizes and detoxifies these inhibitors. Significant transcriptome changes were observed for all inhibitors, with strong induction of genes encoding predicted oxidoreductases, presumably in response to oxidative stress triggered by these chemicals. The detoxification of ferulic acid and vanillin likely occurs via the same catabolic pathway, whereas the main pathway used by *A. oryzae* to alleviate furaldehyde toxicity is the reduction of aldehydes to alcohols. For levulinic acid, active excretion by transporters may be the main tolerance mechanism. Aldehydic inhibitors induced a greater transcriptomic response to oxidative stress than organic acids, as indicated by a metabolic shift to stress response pathways. Several uncharacterized oxidoreductases and transporters were discovered to be differentially regulated by lignocellulosic inhibitors. These genes will be crucial targets for developing robust fungal strains utilizing lignocellulosic side streams as carbon sources.

## Supplementary Information

**Additional file 1:** Additional figures to the article.

**Additional file 2**: Additional tables to the article.

**Additional file 3:** Cluster information.

**Additional file 4:** FASTA file of the fungal amino acid sequences used for the oxidoreductase sequence similarity network.

## Data Availability

The dataset supporting the conclusions of this article is available in NCBI’s Gene Expression Omnibus and is accessible through GEO Series accession number GSE296876 (https://www.ncbi.nlm.nih.gov/geo/query/acc.cgi?acc = GSE296876).
