# Supplementary material for: Transcriptome analysis of Aspergillus oryzae RIB40 under chemical stress reveals mechanisms of adaptation to fungistatic compounds of lignocellulosic side streams
Source: Biotechnol Biofuels Bioprod. 2025 Aug 8;18:89. doi: 10.1186/s13068-025-02688-5 (PMC12335040; doi:10.1186/s13068-025-02688-5)
Supplement: Supplementary file 1 — Additional file 1: Additional figures to the article. [file 13068_2025_2688_MOESM1_ESM.docx]

**Supplementary information**

**Transcriptome analysis of *Aspergillus oryzae* RIB40 under chemical stress reveals mechanisms of adaptation to fungistatic compounds of lignocellulosic side streams**

**Fig. S1.** Principal component analysis (PCA) of the RNA-sequencing samples. The condition is marked by color and time point by shape.

**
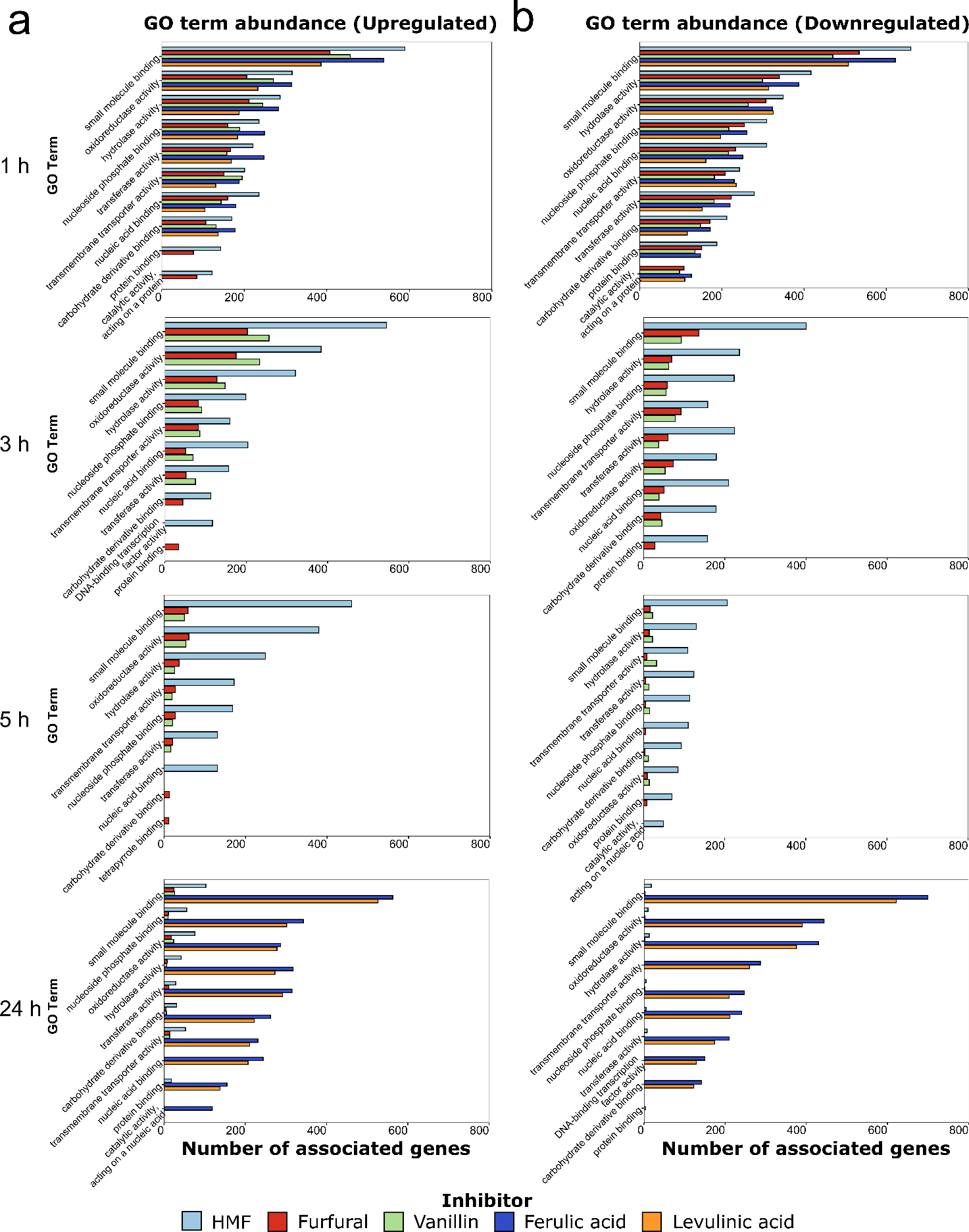
**

**Fig. S2.** Bar charts displaying the most abundant "Molecular function" level 3 GO terms for each condition within the differentially expressed genes (**a**) for upregulated genes (**b**) for downregulated genes.


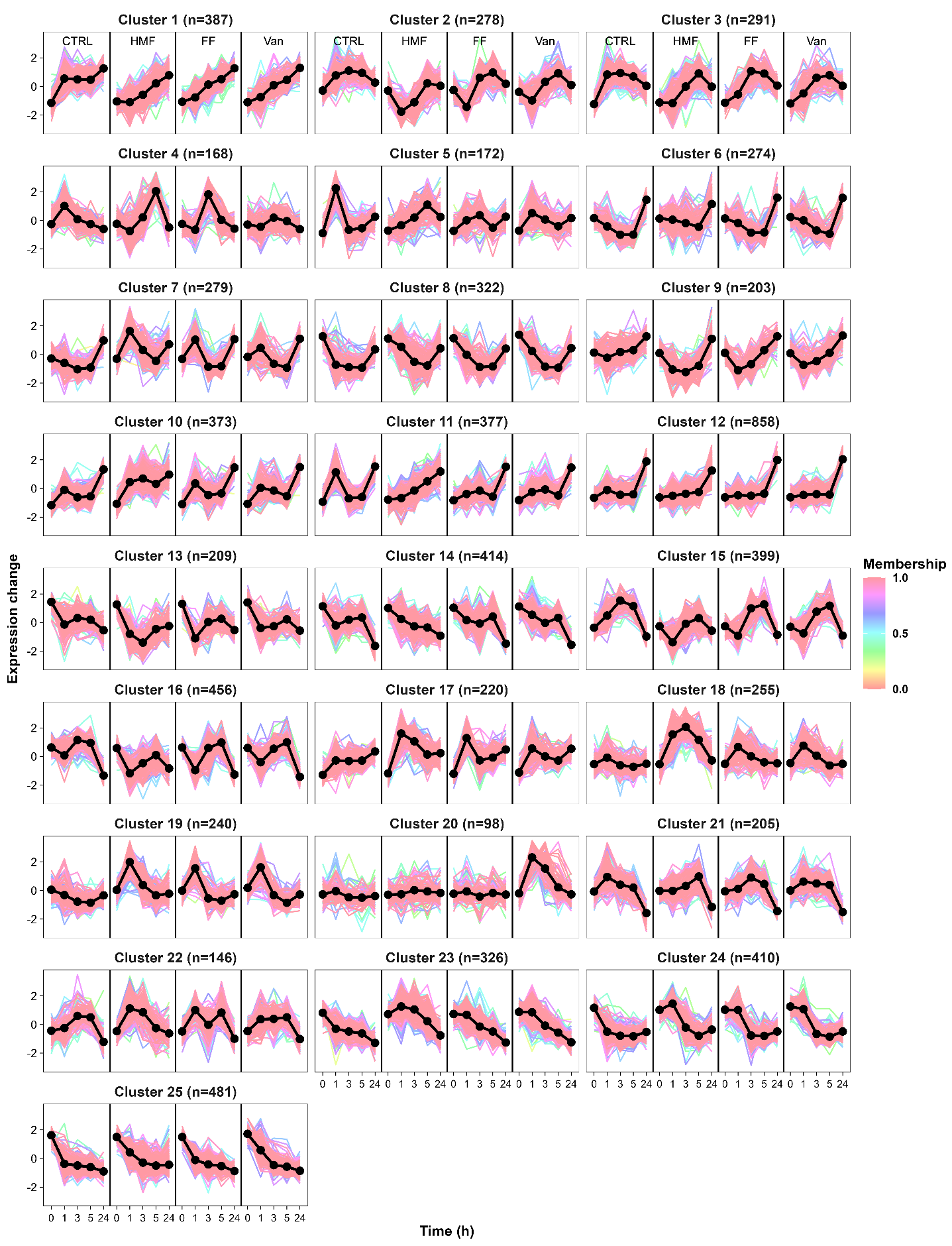


**Fig. S3.** Cluster profiles (25) of the aldehydic compounds. Profiles illustrate the trends in relative gene expression changes over time in both control and treated samples. The black solid line with dots represents the cluster centers, where each dot indicates the average center of biological replicates at a specific time point. Colored lines depict individual gene expression changes, with the color indicating the membership value. Higher values signify a better fit within the cluster. Vertical black lines separate different samples, and n denotes the number of genes in the cluster.


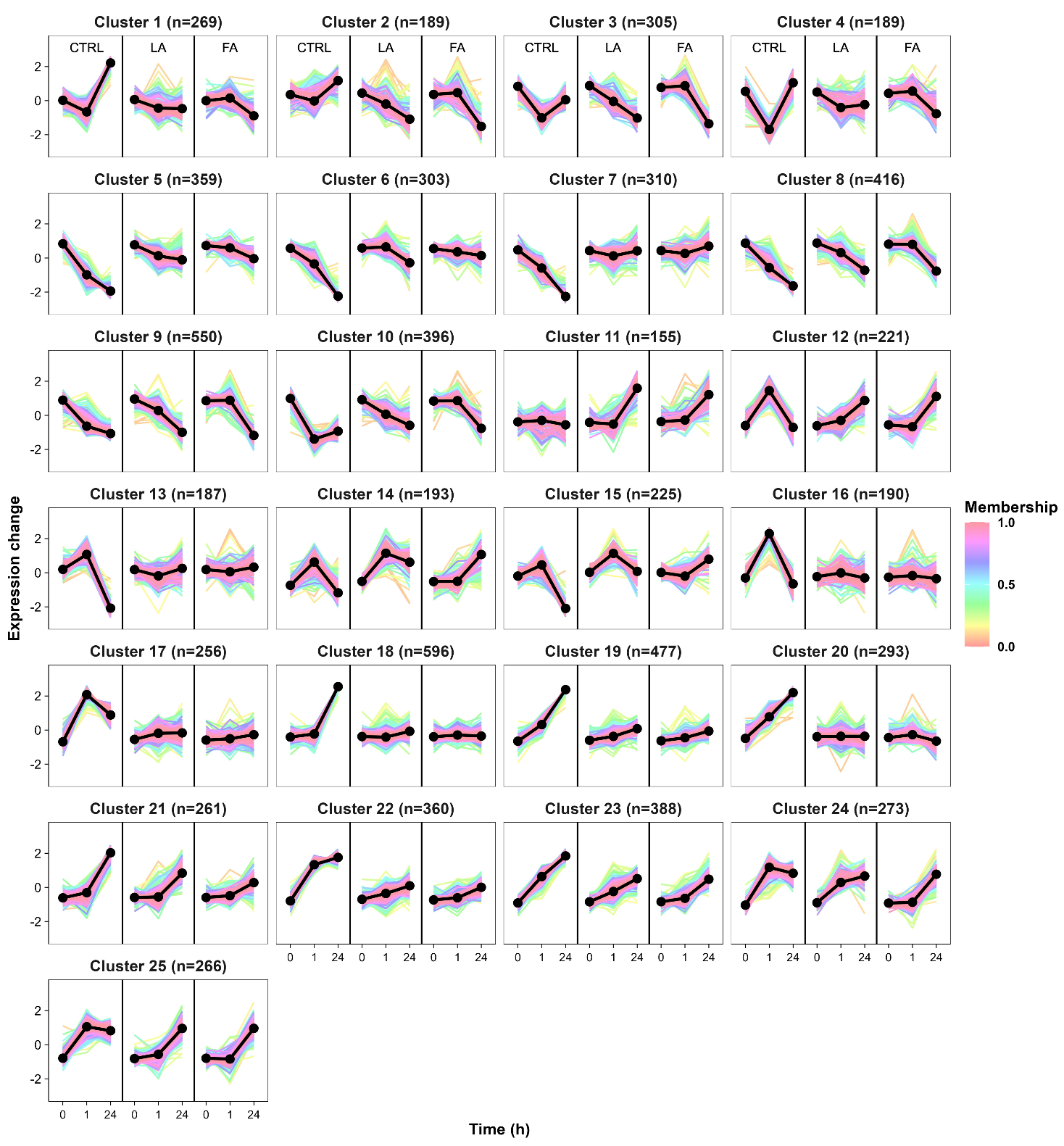
**Fig. S4.** Cluster profiles (25) of the acidic compounds. Profiles illustrate the trends in relative gene expression changes over time in both control and treated samples. The black solid line with dots represents the cluster centers, where each dot indicates the average center of biological replicates at a specific time point. Colored lines depict individual gene expression changes, with the color indicating the membership value. Higher values signify a better fit within the cluster. Vertical black lines separate different samples, and n denotes the number of genes in the cluster.


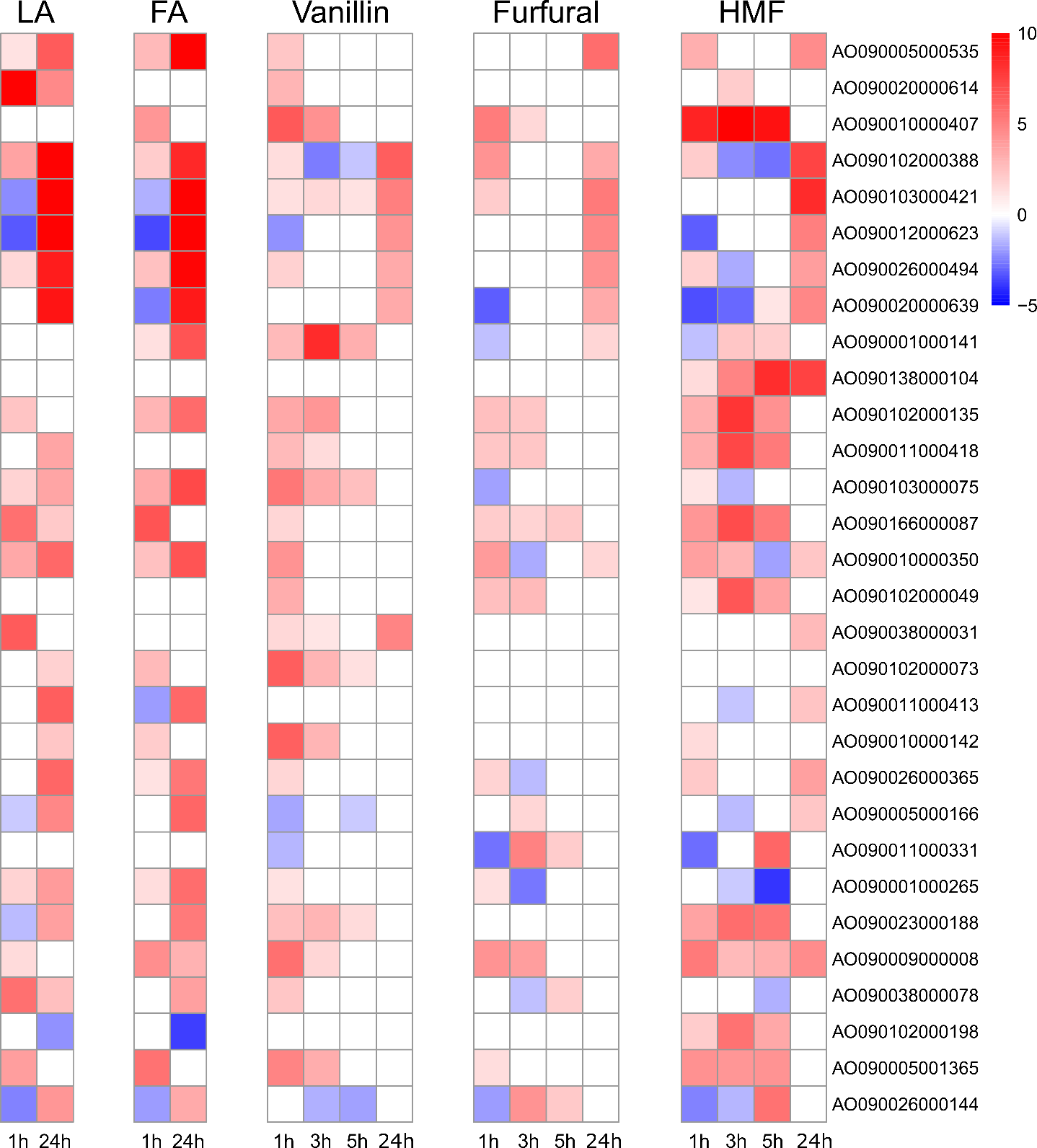


**Fig. S5.** Heatmaps of the most highly upregulated MFS transporters. Heatmaps of LFC values of the 30 most upregulated *A. oryzae* MFS transporter genes under levulinic acid (LA), ferulic acid (FA), vanillin, furfural, and HMF in comparison to the control strain at different time points. LFC values in the heatmaps are capped from -5 to 10.
